# Clinical outcomes of early vs. late extubation following cardiac surgery: a systematic review and meta-analysis

**DOI:** 10.3389/fmed.2026.1913348

**Published:** 2026-08-26

**Authors:** Mohammad S. Dairi, Husna Irfan Thalib, Sariya Khan, Ayesha Jamal, Saeed M. Alghamdi, Abdulelah M. Aldhahir, Abdullah A. Alqarni, Tope Oyelade, Imran Khalid, Sultan Salem Bawazeer, Amir Aziz, Hassan Alwafi

**Affiliations:** 1 Department of Medicine, College of Medicine, Umm Al-Qura University, Makkah, Saudi Arabia; 2 Batterjee Medical College, Jeddah, Saudi Arabia; 3 Respiratory Care Program, Clinical Technology Department, Faculty of Applied Medical Sciences, Umm Al-Qura University, Makkah, Saudi Arabia; 4 Respiratory Therapy Program, Department of Nursing, College of Nursing and Health Sciences, Jazan University, Jazan, Saudi Arabia; 5 Department of Respiratory Therapy, Faculty of Medical Rehabilitation Sciences, King Abdulaziz University, Jeddah, Saudi Arabia; 6 School of Medicine, Keele University, Keele, Stoke-on-Trent, United Kingdom; 7 Division of Medicine, University College London, London, United Kingdom; 8 Alfaisal University, Riyadh, Saudi Arabia; 9 King Faisal Specialist Hospital and Research Center, Jeddah, Saudi Arabia; 10 Department of Surgery, College of Medicine, Al-Qunfudah, Umm Al-Qura University, Makkah, Saudi Arabia; 11 Health Informatics Specialist Digitization Department Dallah Health Co, Riyadh, Saudi Arabia; 12 Department of Pharmacology and Toxicology, College of Medicine, Umm Al-Qura University, Makkah, Saudi Arabia

**Keywords:** cardiac surgery, early extubation, late extubation, mechanical ventilalion, mortality

## Abstract

**Objective:**

This systematic review and meta-analysis evaluated clinical outcomes associated with earlier vs. later extubation in adults undergoing cardiac surgery.

**Methods:**

A systematic review and meta-analysis were conducted following PRISMA guidelines, with the study registered in PROSPERO. We searched PubMed, Scopus, Web of Science, CENTRAL, and EBSCO from inception until April 2025. Two reviewers independently screened studies. We included observational and interventional studies comparing early vs. late extubation following cardiac surgery in adult patients. Primary outcomes were all-cause mortality, length of intensive care unit (ICU) stay, length of hospital stay, reintubation, postoperative pneumonia, and postoperative renal failure requiring continuous renal replacement therapy (CRRT). Secondary outcomes included mediastinal bleeding requiring re-exploration, tracheostomy, and hospital or ICU readmission. Risk of bias in randomized trials was assessed using the Cochrane Risk of Bias tool, while the Newcastle-Ottawa Scale (NOS) was used for observational studies. Data synthesis was performed using Review Manager version 5.4. For continuous outcomes, pooled effect estimates were expressed as mean differences (MDs). For dichotomous outcomes, pooled odds ratios (ORs) with corresponding 95% CIs were calculated. Random-effects models were used as the primary analysis. A subgroup analysis based on extubation threshold ( ≤ 6 h or >6 h) was conducted. Statistical heterogeneity was assessed using the Chi-square test and the *I*^2^ statistic.

**Results:**

A total of 237 records were identified, of which only 19 studies (18 observational and one randomized trial) were included in this meta-analysis. Earlier extubation was associated with lower odds of mortality (OR 0.09, 95% CI 0.04–0.23), reintubation (OR 0.22, 95% CI 0.07–0.70), postoperative pneumonia (OR 0.20, 95% CI 0.09–0.45), renal failure requiring CRRT (OR 0.10, 95% CI 0.06–0.17), postoperative tracheostomy (OR 0.01, 95% CI: 0.00–0.03), and hospital or ICU readmission (OR 0.25, 95% CI 0.07–0.86).

**Conclusions:**

In adults undergoing cardiac surgery, earlier extubation was associated with favorable postoperative outcomes including lower mortality, shorter ICU and hospital stays, and lower rates of reintubation, postoperative pneumonia, tracheostomy, CRRT, and hospital or ICU readmission. However, future randomized trials and prospective studies are warranted as the available evidence was predominantly observational and clinically heterogeneous.

**Systematic review registration:**

https://www.crd.york.ac.uk/PROSPERO/view/CRD420261361735, identifier: CRD420261361735.

## Introduction

Cardiac surgery is one of the most resource- and technique-intensive disciplines of modern medicine, with over one million annual operations worldwide, including CABG, valve repair or replacement, and congenital defect correction (1, 2). Many of these cases necessitate CPB, thus imposing extreme physiological stress on the body that demands vigilant attention to perioperative and postoperative care, particularly when it comes to the provision of respiratory support. Although mechanical ventilation is largely unavoidable in maintaining stability and ensuring adequate oxygenation during the immediate postoperative period, it is not without risk. Indeed, extended intubation is associated with an increase incidence of ventilator-associated pneumonia (VAP), extended stay in both the ICU and hospital, increased healthcare expenditure, and increased morbidity and mortality (3–5).

In response, fast-track or early extubation has gained increasing interest as a strategy to accelerate postoperative recovery and optimize resource utilization. A 2024 joint consensus statement from the Enhanced Recovery After Surgery (ERAS) Cardiac Society, ERAS International Society, and the Society of Thoracic Surgeons (STS) states that structured strategies facilitating extubation within 6 h after surgery are safe and may hasten recovery after routine elective cardiac procedures (6). This contemporary benchmark does not eliminate the substantial historical variation in definitions, which has ranged from extubation in the operating room to thresholds of 24–48 h or longer (33, 34).

Early-extubation policies vary among institutions, surgeons, and anesthesiologists despite their potential advantages, partly because of concerns about premature extubation, reintubation, hypoxemia, hemodynamic compromise, and respiratory failure. The absence of uniform criteria and time thresholds has also limited comparability across studies. Comparative studies have evaluated ICU stay, reintubation, mortality, and other postoperative outcomes, but their findings remain susceptible to differences in patient selection, procedural complexity, and institutional protocols (7, 8).

Importantly, the decision to perform early extubation is not randomized in routine clinical practice but is based on careful assessment of perioperative stability. Patients selected for early extubation are typically younger, have fewer comorbidities, shorter cardiopulmonary bypass times, less intraoperative bleeding, lower vasopressor requirements, and fewer postoperative complications. Consequently, early extubation may represent a marker of favorable postoperative recovery rather than an independent intervention responsible for improved clinical outcomes. This distinction should be considered when interpreting evidence derived primarily from observational studies.

The key determinants will include age, underlying comorbidities (for example, chronic kidney disease, heart failure), intraoperative course (for example, CPB duration, bleeding), and type of surgical procedure (isolated CABG vs. combined valve-CABG). A systematic review and meta-analysis of strong comparative evidence is therefore required to establish whether early extubation is uniformly beneficial as compared with the conventional or delayed strategy in heterogeneous patient populations.

The aim of this systematic review and meta-analysis was to synthesize the available evidence comparing early vs. late extubation in patients undergoing cardiac surgery. The primary objective was to evaluate the effect of extubation timing on ICU length of stay, hospital length of stay, mortality, reintubation, postoperative pneumonia, and postoperative renal failure requiring continuous renal replacement therapy (CRRT). Secondary outcomes included mediastinal bleeding requiring re-exploration, tracheostomy, and hospital or ICU readmission. By integrating evidence from diverse clinical settings and study designs, this review aimed to inform perioperative practice and support evidence-based recommendations regarding optimal extubation timing following cardiac surgery.

## Methodology

### Protocol approval and registration

This systematic review and meta-analysis were conducted in accordance with the Preferred Reporting Items for Systematic Reviews and Meta-Analyses (PRISMA) 2020 guidelines. A completed PRISMA Checklist is available in Supplementary Table S1. The study protocol was prospectively registered in the International Prospective Register of Systematic Reviews (PROSPERO) under registration no. CRD42024628197. As the review involved secondary data collection from already published studies, no ethical approval or informed consent was required.

### Search strategy

A comprehensive literature search was conducted across five electronic databases: PubMed, Scopus, Web of Science, Cochrane Central Register of Controlled Trials (CENTRAL), and EBSCO. The search included studies published up to April 2025 and was designed to identify research examining outcomes of early vs. late extubation following cardiac surgery. The search terms used were as follows: (“cardiac surgery” OR “cardiopulmonary bypass” OR “CABG” OR “valve replacement” OR “open-heart surgery”) AND (“early extubation” OR “fast-track extubation” OR “ultra-fast track” OR “delayed extubation” OR “late extubation”) AND (“ICU stay” OR “ventilator time” OR “mortality” OR “postoperative outcomes” OR “complications” OR “length of hospital stay”) AND (“randomized controlled trial” OR “cohort study” OR “observational study” OR “clinical trial”). Boolean operators and MeSH terms were applied to optimize sensitivity and specificity. Reference lists of all eligible full-text articles and relevant systematic reviews were manually searched to identify additional studies not captured by database searches.

### Study selection criteria

Studies were included if they were published in English and involved adults aged 18 years or older undergoing cardiac surgery. Eligible studies directly compared earlier vs. later extubation and reported at least one prespecified clinical outcome. Exclusively pediatric studies were excluded. Mixed-age studies were eligible only when adult data were separately extractable. Because the protocol preceded publication of the 2024 joint ERAS–STS consensus statement and no universal definition existed at registration, study-specific extubation definitions were retained for the primary synthesis. For sensitivity analyses, studies were categorized according to whether the early-extubation threshold was 6 h or less or greater than 6 h. This category was intended to distinguish contemporary fast-track thresholds from broader definitions and was not treated as an exact ≤ 6-vs.->6-h comparison when an individual study used a different cutoff. Randomized trials, prospective or retrospective cohorts, and case–control studies were eligible. Studies without a comparator, without a clear timing definition, case reports, case series, reviews, editorials, conference abstracts, and non-English reports were excluded.

### Screening

All references retrieved through the search strategy were imported into Rayyan, a web-based tool for managing systematic review workflows. Two reviewers independently screened all titles and abstracts to identify potentially relevant studies. Full texts of selected articles were then reviewed independently to assess eligibility based on the inclusion and exclusion criteria. Disagreements during either phase were resolved through consensus, and if necessary, arbitration by a third reviewer.

### Data extraction and risk of bias assessment

Primary outcome measures included ICU length of stay, hospital length of stay, mortality, reintubation, postoperative pneumonia, and postoperative renal failure requiring CRRT. Secondary outcomes included mediastinal bleeding requiring re-exploration, tracheostomy, and hospital or ICU readmission. Baseline characteristics, including chronic kidney disease and congestive heart failure with reduced ejection fraction, were extracted when available. Risk of bias in randomized trials was assessed using the Cochrane Risk of Bias 2 tool, whereas the Newcastle–Ottawa Scale was used for observational studies. Risk-of-bias judgments were incorporated into interpretation and certainty assessment; studies were not automatically excluded solely because of an unfavorable quality rating.

### Outcome measures

The primary outcomes assessed were all-cause mortality, length of intensive care unit (ICU) stay, length of hospital stay, reintubation, postoperative pneumonia, and postoperative renal failure requiring continuous renal replacement therapy (CRRT). Secondary outcomes included mediastinal bleeding requiring re-exploration, tracheostomy, and hospital or ICU readmission. These outcomes were selected to evaluate both the clinical effectiveness and safety of early extubation following cardiac surgery.

### Data synthesis and statistical analysis

Data synthesis was performed using Review Manager (RevMan) version 5.4. For continuous outcomes, pooled effect estimates were expressed as mean differences (MDs) or standardized mean differences (SMDs) with 95% confidence intervals (CIs), as appropriate. For dichotomous outcomes, pooled odds ratios (ORs) with corresponding 95% CIs were calculated. Random-effects models were used as the primary analytical approach to account for anticipated clinical and methodological heterogeneity across studies. Fixed-effect models were applied in analyses with negligible statistical heterogeneity or as appropriate based on the distribution and consistency of the included studies. Statistical heterogeneity was assessed using the Chi-square test and quantified using the *I*^2^ statistic, with values above 50% considered moderate to substantial. Sensitivity subgroup analyses categorized studies by the threshold used to define earlier extubation: (1) an early-extubation threshold of 6 h or less and (2) a threshold greater than 6 h. Studies using 4-h thresholds were therefore included in the first category but were not interpreted as exact 6-h comparisons. Differences between subgroups were evaluated using RevMan's test for subgroup differences. Random-effects models were preferred because of expected variation in operation type, procedural complexity, institutional protocols, and patient selection. Formal meta-regression was not undertaken because too few studies reported consistent procedure-level and patient-level covariates.

## Results

**Figure 1:**
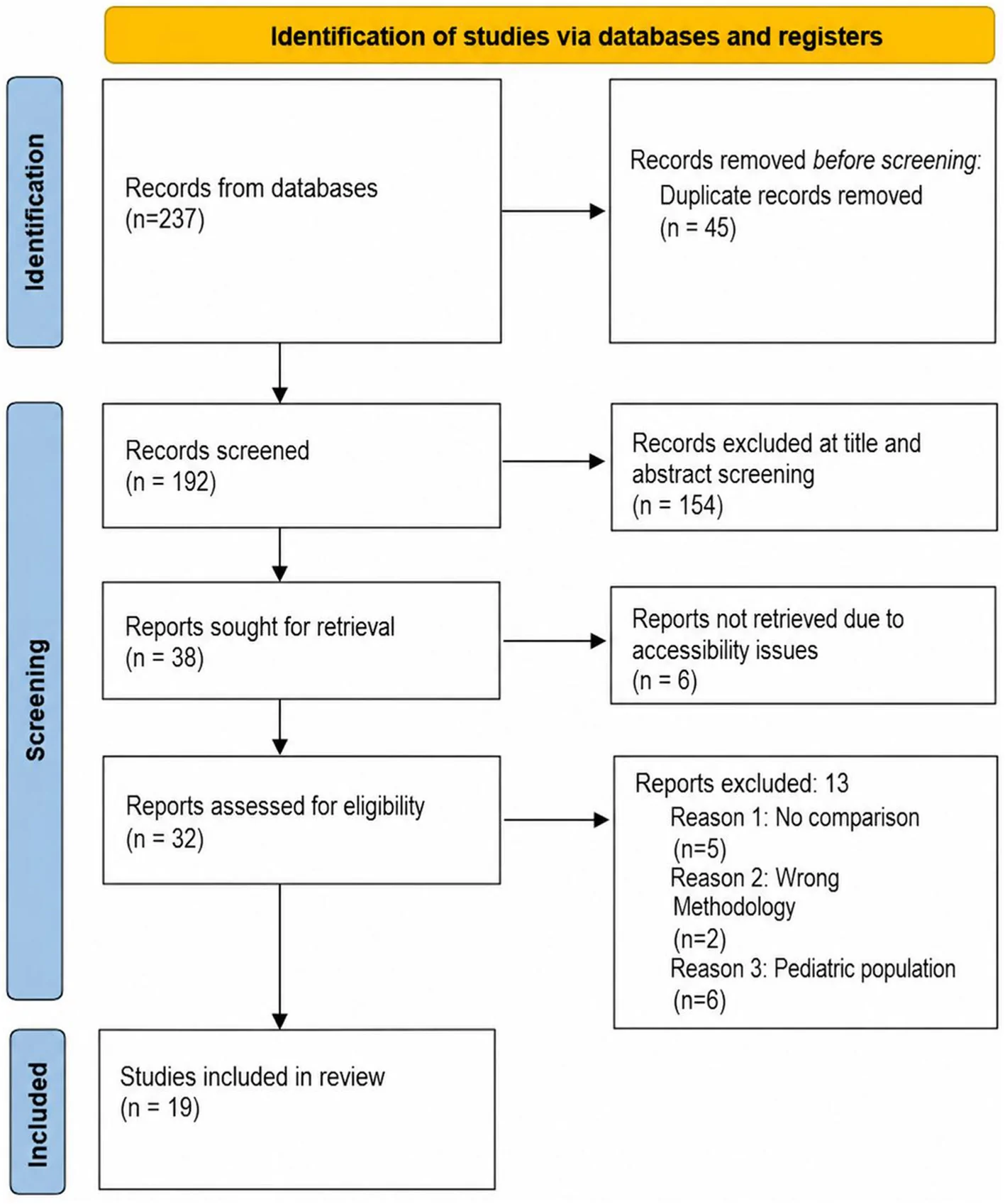
PRISMA flowchart.

A total of 237 records were identified through database searches. After removal of 45 duplicates, 192 records were screened and 154 were excluded at title and abstract screening. Thirty-eight reports were sought for retrieval, of which six could not be obtained. Thirty-two full-text reports were assessed. Thirteen were excluded: five lacked a comparison group, two had an incompatible design, and six were exclusively pediatric. Nineteen adult studies were therefore, included in the systematic review and meta-analysis (Figure 1).

Table 1 summarizes the characteristics of studies included in the systematic review. The evidence base comprised several observational studies, and one randomized controlled trial. The studies were conducted across a wide range of countries and included heterogeneous adult cardiac surgical populations, such as patients undergoing coronary artery bypass grafting, valve surgery, combined CABG–valve procedures, aortic surgery, ventricular assist device implantation, heart transplantation, and selected adult congenital cardiac procedures. Definitions of earlier and later extubation varied considerably across studies, ranging from extubation within 4–6 h after surgery to thresholds of 24, 48, or 72 h, and in some studies extending to several days of mechanical ventilation. This variability reflects differences in surgical complexity, institutional fast-track protocols, and the clinical characteristics of the included populations. Sample sizes also varied substantially, from small single-center cohorts to studies including more than 1,000 participants. The marked heterogeneity in study design, operative population, and extubation thresholds should be considered when interpreting the pooled findings.

**Table 1:** General characteristics of the included adult studies.

References	Country	Study design	Earlier-extubation definition	Later-extubation definition	Cardiac surgery/population	Total N	Mean age, years
Rashid (17)	Pakistan	Retrospective cohort	< 4 h	>4 h	CABG, valve replacement, and atrial septal repair	NR	NR
Camp (18)	USA	Retrospective cohort	< 6 h	>6 h	CABG, valve surgery, and combined CABG–valve surgery	NR	NR
Thanavaro (19)	USA	Retrospective cohort	< 48 h	>48 h	CABG, valve surgery, and combined CABG–valve surgery	NR	NR
Fischbach (20)	Germany	Retrospective cohort	< 6 h	>6 h	Surgical aortic valve replacement	NR	NR
Zettervall (21)	USA	Retrospective cohort	Operating-room extubation or < 12 h postoperatively	>12 h after EVAR or >24 h after open repair	Open or endovascular abdominal aortic aneurysm repair	NR	NR
Naughton (22)	UK	Sequential cohort analysis	< 6 h	>6–24 h, >24–48 h, or >48 h	OPCAB and complex cardiac surgery, including valve, aortic, combined, and redo CABG procedures	NR	NR
Yigit (23)	Turkey	Observational cross-sectional study	≤ 8 h	>8 h	CABG, valve surgery, aortic dissection surgery, and LVAD/BIVAD implantation	NR	NR
Aksoy (24)	Turkey	Retrospective cohort	< 24 h	>24 h	Cardiac surgery with cardiopulmonary bypass	NR	NR
Farooq (25)	Pakistan	Analytical cross-sectional study	≤ 4 h	>4 h	Isolated CABG, isolated valve replacement, and non-complex adult congenital cardiac procedures	NR	NR
Amini (26)	Iran	Retrospective cohort	< 6 h or 7–24 h	>24 h	Isolated CABG	NR	60.24 ± 10.57
Maisat (10)	Thailand	Retrospective cohort	≤ 48 h	>48 h	Acute type A aortic dissection surgery, including ascending aortic or total arch replacement with additional procedures	239	NR
Shirzad (11)	Iran	Observational cohort	< 24 h	≥24 h	Heart valve surgery, including aortic, mitral, and combined valve replacement	1,056	48.43 ± 13.7
Trouillet (27)	France	Prospective observational cohort	≤ 72 h	>72 h	CABG, valve, combined CABG–valve, transplantation, and other complex cardiovascular surgery	163	63.97 ± 14.99
Papathanasiou (9)	Germany	Retrospective observational study	≤ 7 days of ventilation	>7 days of ventilation	LVAD implantation	139	57.5 ± 10.5
Chan (28)	USA	Retrospective review	≤ 6 h	>6 h	CABG, valve, combined CABG–valve, and other cardiac procedures	1,581	68
Kern (29)	Germany	Prospective cohort	< 48 h	>48 h	CABG, valve, and combined cardiac surgery	687	62.3 early; 66.2 late
Affronti (30)	Italy	Retrospective observational study	Tracheostomy on or before day 14	Tracheostomy after day 14	Cardiac surgical patients requiring prolonged mechanical ventilation	112	NR
Trouillet (31)	France	Randomized controlled single-center trial	Early percutaneous tracheotomy	Prolonged intubation	Mechanically ventilated patients after CABG, valve, combined, transplant, or other cardiovascular surgery	216	NR
Reddi (32)	Australia	Retrospective cohort	< 18 h	≥18 h	CABG and/or valve surgery	NR	NR

Supplementary Table S2 summarizes the variability in adult operative populations and extubation definitions. Earlier-extubation definitions ranged from operating-room extubation to thresholds extending over several days, while delayed or prolonged ventilation definitions also varied. This heterogeneity reflects distinct adult cardiac procedures with different expected postoperative ventilatory trajectories. The subgroup based on an early-extubation threshold of 6 h or less was therefore treated as a clinically anchored sensitivity analysis rather than as a universal definition applicable to every procedure or study.

### Risk of bias

**Figure 2:**
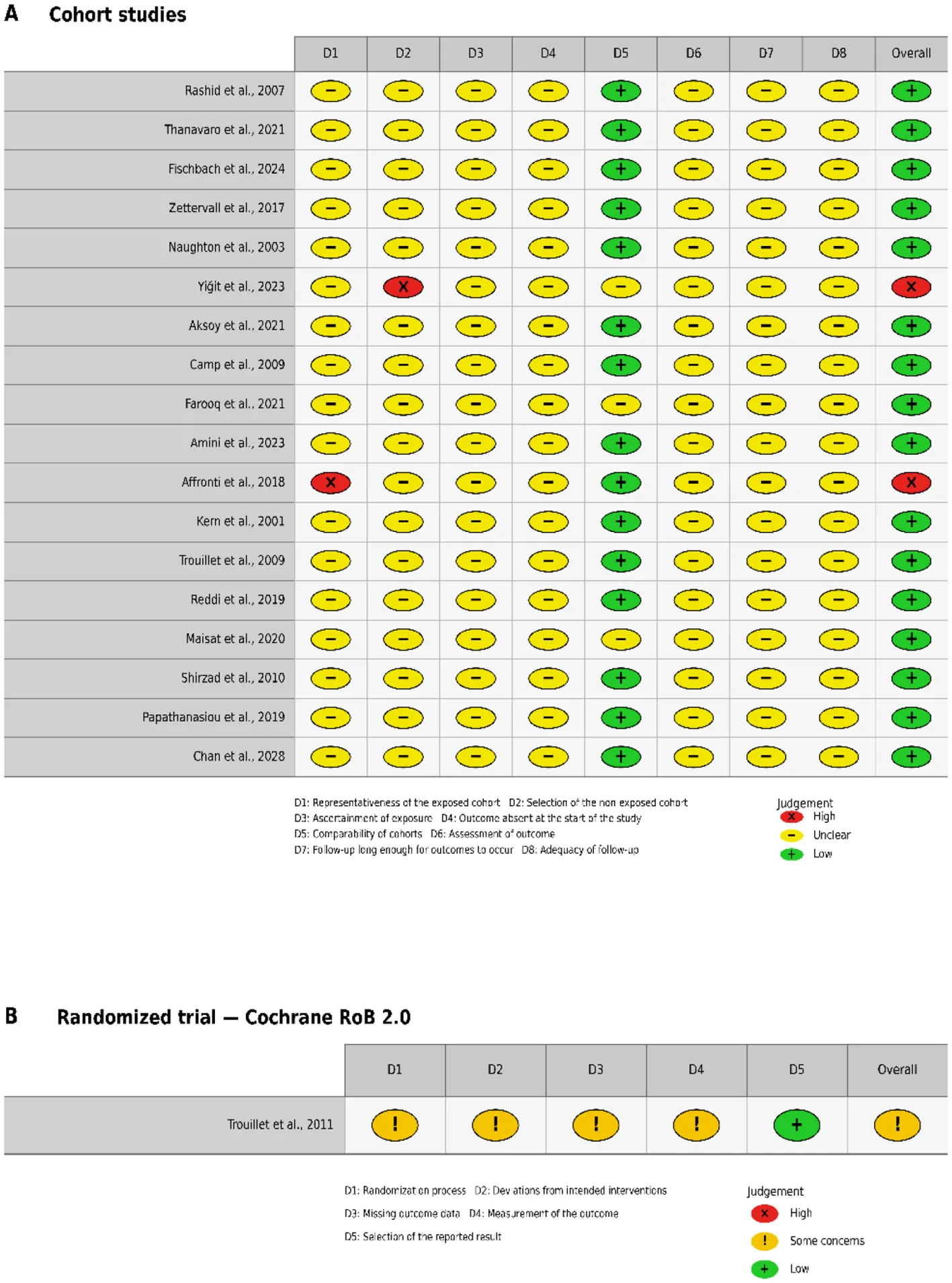
Risk-of-bias assessment of the included studies. Panel **(A)** shows the Newcastle–Ottawa scale assessment of the 18 cohort studies, most of which were judged to have a low overall risk of bias; Yigit et al. (23) and Affronti et al. (30) were rated as high risk. Panel **(B)** presents the Cochrane RoB 2 assessment of Trouillet et al. (31), which was judged have some concerns overall.

The methodological quality of the included studies was assessed using design-specific risk-of-bias tools. The 18 cohort studies were evaluated across the Newcastle–Ottawa Scale domains, with most receiving an overall low-risk judgement, although unclear ratings were noted where reporting was insufficient. Two cohort studies were judged to have a high overall risk of bias because of concerns within individual methodological domains. The single randomized controlled trial was assessed using the Cochrane Risk of Bias 2 tool and was judged to have some concerns overall, mainly across the randomization process, deviations from intended interventions, missing outcome data, and outcome measurement domains (Figure 2).

### Mortality

**Figure 3:**
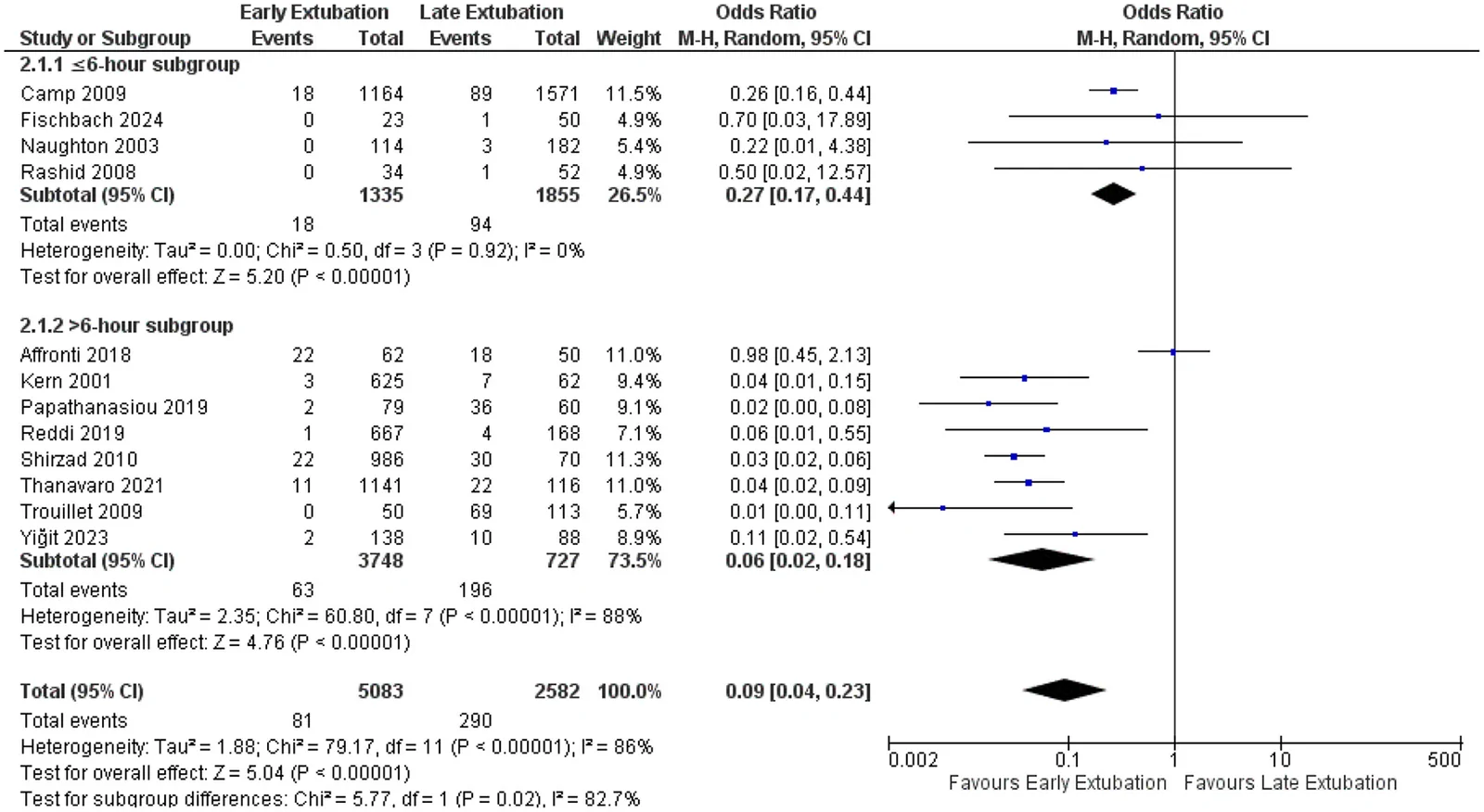
Association between earlier vs. later extubation and all-cause mortality following adult cardiac surgery, stratified by extubation-threshold category.

Twelve studies contributed mortality data (Figure 3). Earlier extubation was associated with lower odds of mortality (OR 0.09, 95% CI 0.04–0.23; *p* < 0.00001), although heterogeneity was substantial (*I*^2^ = 86%). When stratifying the analysis according to the early-extubation threshold, studies using a threshold of ≤ 6 h showed a pooled OR 0.27 (95% CI 0.17–0.44; *I*^2^ = 0%) while whereas studies using thresholds >6 h showed a pooled OR 0.06 (95% CI 0.02–0.18; *I*^2^ = 88%). The subgroup difference was statistically significant (*p* = 0.02).

### Length of ICU stays

**Figure 4:**
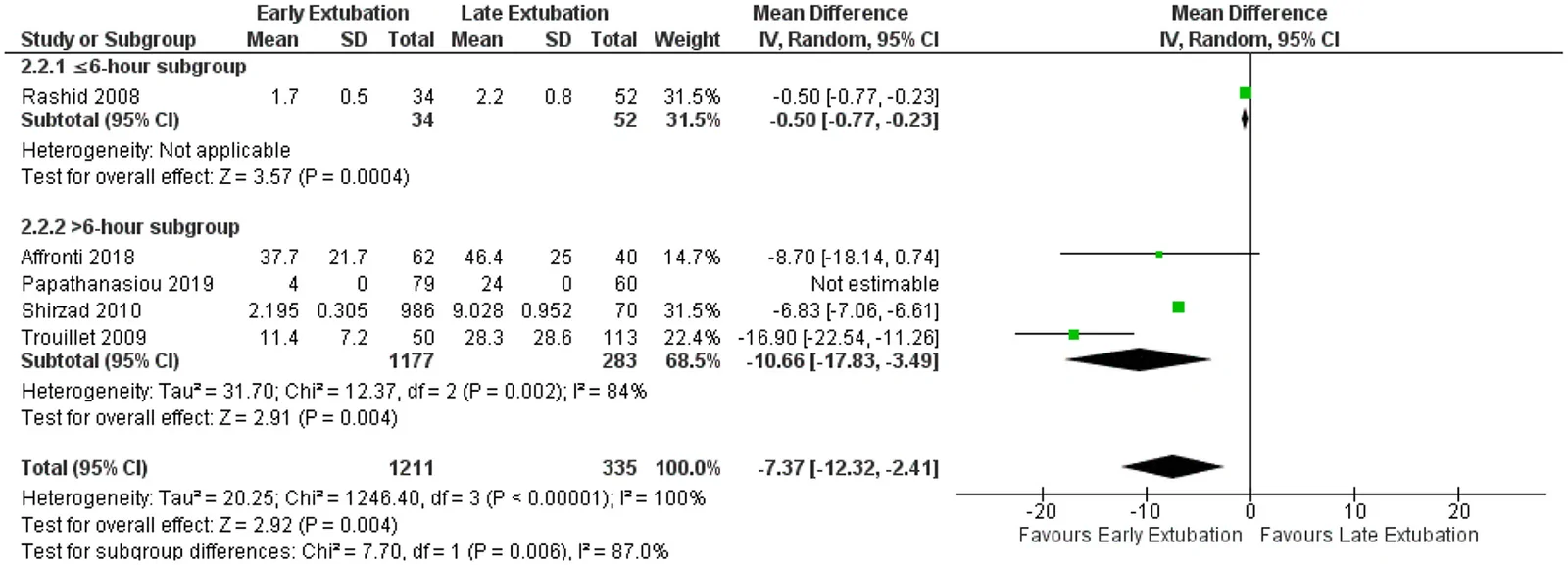
Difference in ICU length of stay (in days) between earlier and later extubation groups following adult cardiac surgery, stratified by extubation-threshold category.

Four estimable studies contributed ICU length-of-stay data (Figure 4). Earlier extubation was associated with a shorter ICU stay (MD −7.37, 95% CI −12.32 to −2.41; *p* = 0.004), but heterogeneity was extreme (*I*^2^ = 100%). The study using an early-extubation threshold of ≤ 6 h reported an MD of −0.50 (95% CI −0.77 to −0.23), whereas studies using thresholds >6 h produced an MD of −10.66 (95% CI −17.83 to −3.49; *I*^2^ = 84%). The subgroup difference was significant (*p* = 0.006).

### Length of hospital stay (days)

**Figure 5:**
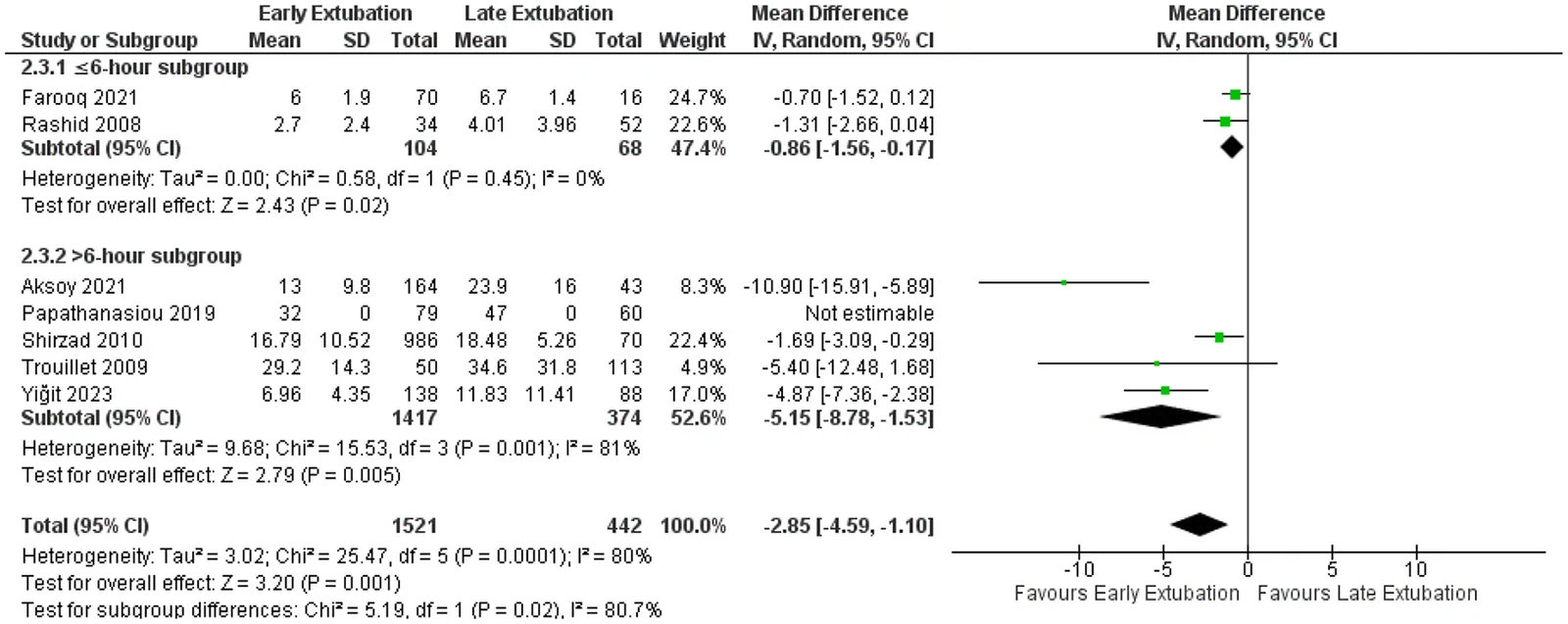
Difference in hospital length of stay between earlier and later extubation groups following adult cardiac surgery, stratified by extubation-threshold category.

Six studies provided estimable hospital length-of-stay data (Figure 5). Earlier extubation was associated with a shorter hospital stay (MD −2.85 days, 95% CI −4.59 to −1.10; *p* = 0.001), with considerable heterogeneity (*I*^2^ = 80%). The pooled MD was −0.86 days (95% CI −1.56 to −0.17; *I*^2^ = 0%) among studies using threshold of ≤ 6 h and −5.15 days (95% CI −8.78 to −1.53; *I*^2^ = 81%) among studies using thresholds >6 h. The subgroup difference was significant (*p* = 0.02). Papathanasiou et al. (9) was not estimable because a usable measure of variability was unavailable for one group.

### Postoperative reintubation

**Figure 6:**
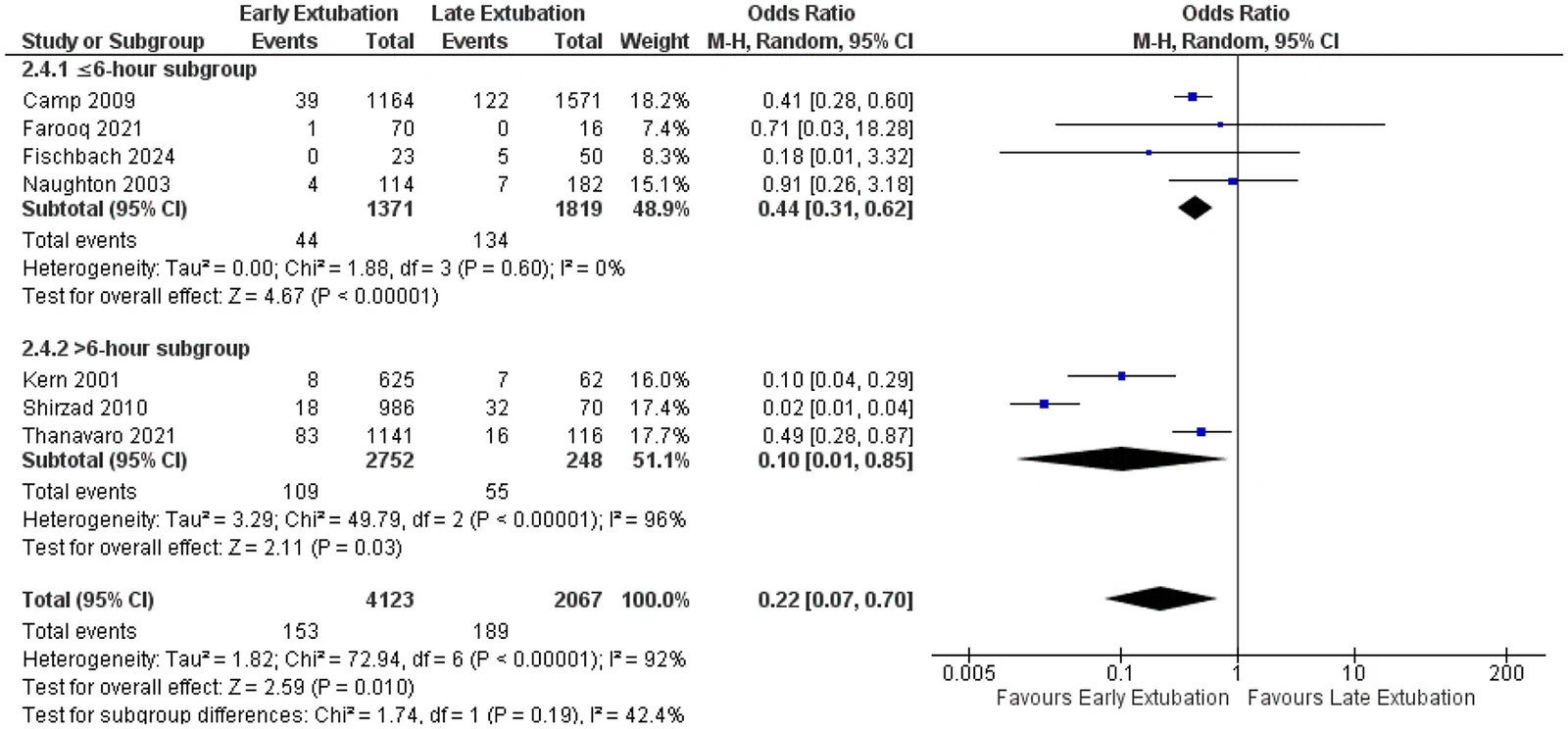
Association between earlier vs. later extubation and postoperative reintubation following adult cardiac surgery, stratified by extubation-threshold category.

Seven studies reported postoperative reintubation (Figure 6). Earlier extubation was associated with lower odds of reintubation (OR 0.22, 95% CI 0.07–0.70; *p* = 0.01), with substantial heterogeneity (*I*^2^ = 92%). The pooled estimate was OR 0.44 (95% CI 0.31–0.62; *I*^2^ = 0%) among studies using threshold of ≤ 6 h and OR 0.10 (95% CI 0.01–0.85; *I*^2^ = 96%) among studies using thresholds >6 h. The subgroup difference was not significant (*p* = 0.19).

### Postoperative pneumonia

**Figure 7:**
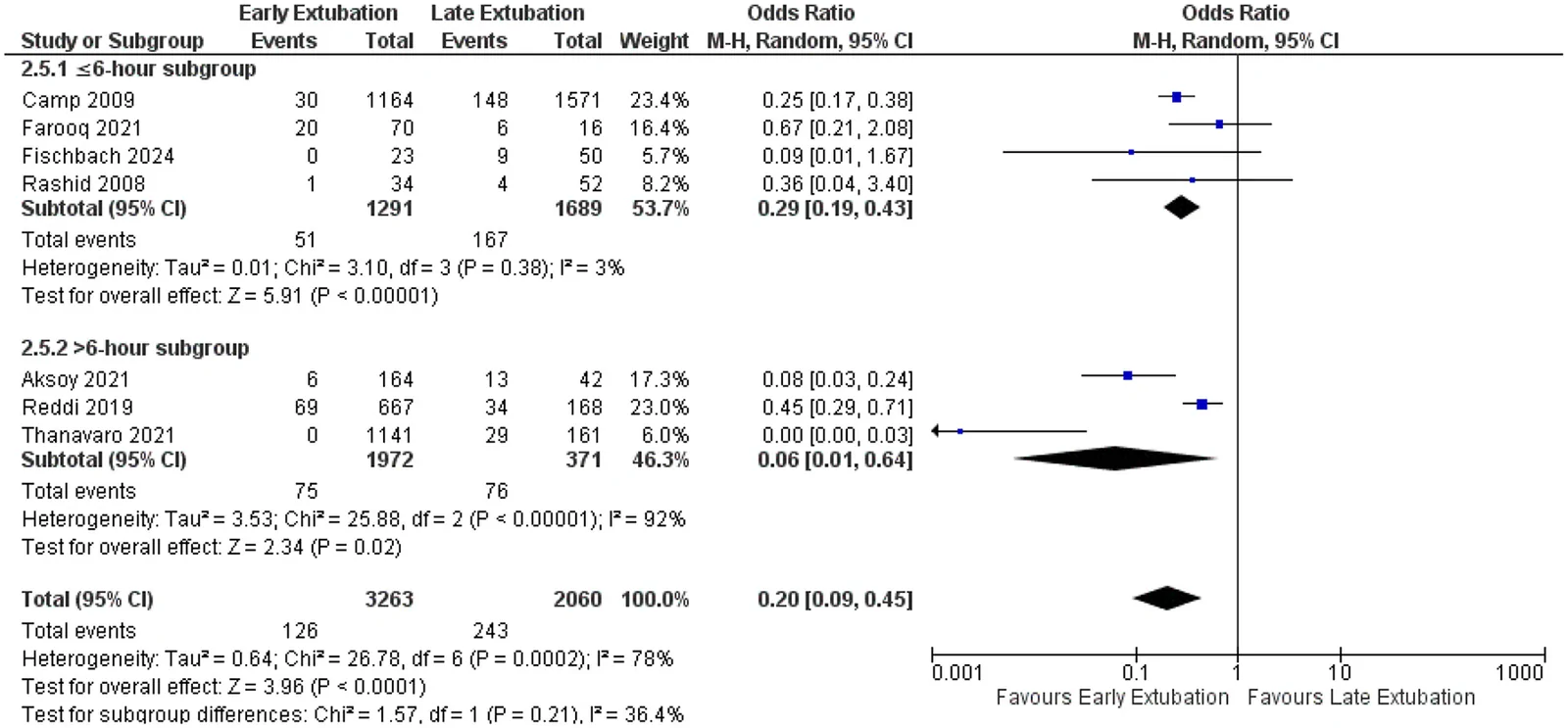
Association between earlier vs. later extubation and postoperative pneumonia following adult cardiac surgery, stratified by extubation-threshold category.

Seven studies contributed postoperative pneumonia data (Figure 7). Earlier extubation was associated with lower odds of pneumonia (OR 0.20, 95% CI 0.09–0.45; *p* < 0.0001), although heterogeneity was considerable (*I*^2^ = 78%). The pooled estimate was OR 0.29 (95% CI 0.19–0.43; *I*^2^ = 3%) among studies using threshold of ≤ 6 h and OR 0.06 (95% CI 0.01–0.64; *I*^2^ = 92%) among studies using thresholds >6 h. The subgroup difference was not significant (*p* = 0.21).

### Postoperative continuous renal replacement therapy (CRRT)

**Figure 8:**
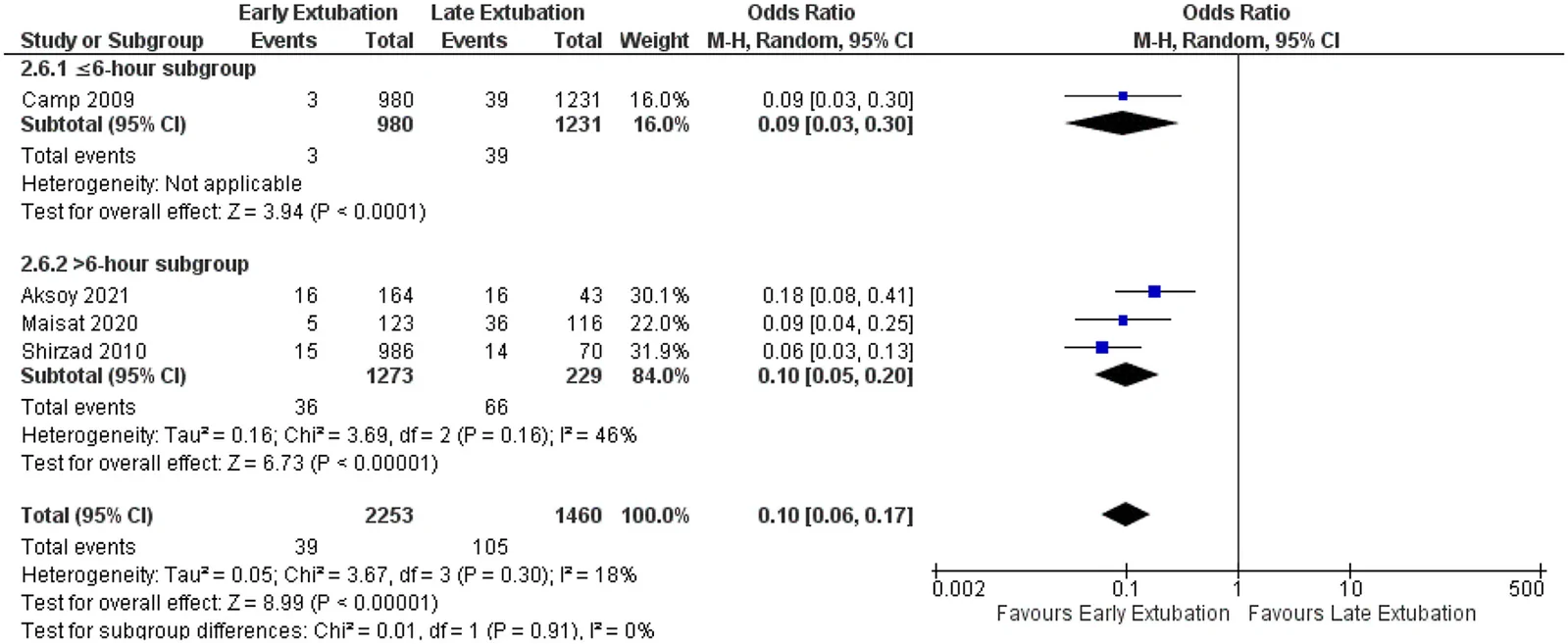
Association between earlier vs. later extubation and postoperative renal failure requiring continuous renal replacement therapy following adult cardiac surgery, stratified by extubation-threshold category.

Four studies reported renal failure requiring CRRT (Figure 8). Earlier extubation was associated with lower odds of CRRT use (OR 0.10, 95% CI 0.06–0.17; *p* < 0.00001), with low statistical heterogeneity (*I*^2^ = 18%). The estimate was OR 0.09 (95% CI 0.03–0.30) in the threshold of ≤ 6 h category and OR 0.10 (95% CI 0.05–0.20; *I*^2^ = 46%) among studies using thresholds >6 h. There was no subgroup difference (*p* = 0.91). Because postoperative renal failure can itself delay extubation, reverse causation is a major concern.

### Mediastinal bleeding

**Figure 9:**
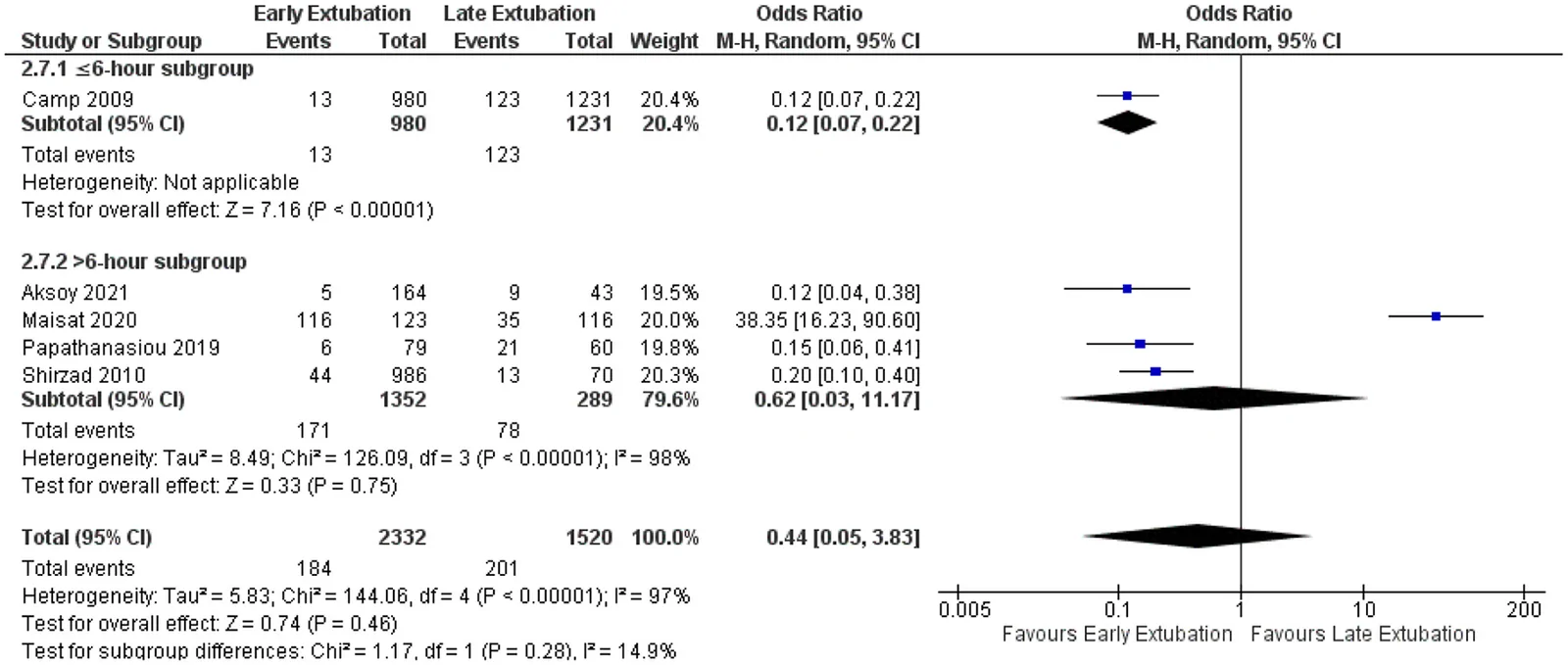
Association between earlier vs. later extubation and mediastinal bleeding requiring surgical re-exploration following adult cardiac surgery, stratified by extubation-threshold category.

Five studies reported mediastinal bleeding or re-exploration (Figure 9). The pooled analysis did not demonstrate a significant association with extubation timing (OR 0.44, 95% CI 0.05–3.83; *p* = 0.46), and heterogeneity was extreme (*I*^2^ = 97%). The study using a threshold of ≤ 6 h reported OR 0.12 (95% CI 0.07–0.22), whereas the pooled estimate among studies using thresholds >6 h was inconclusive (OR 0.62, 95% CI 0.03–11.17; *I*^2^ = 98%). The subgroup difference was not significant (*p* = 0.28).

### Hospital or ICU readmission

**Figure 10:**
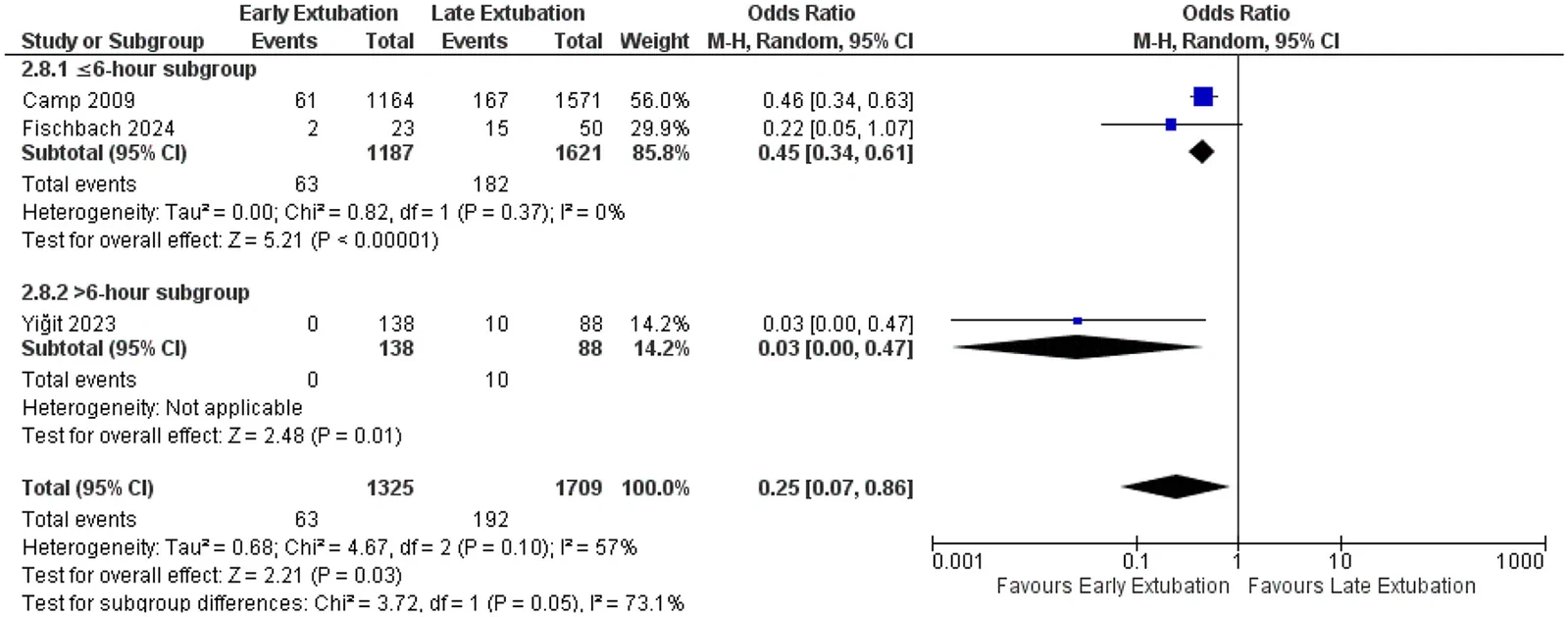
Association between earlier vs. later extubation and hospital or ICU readmission following adult cardiac surgery, stratified by extubation-threshold category.

Three studies reported hospital or ICU readmission (Figure 10). Earlier extubation was associated with lower odds of readmission (OR 0.25, 95% CI 0.07–0.86; *p* = 0.03), with moderate heterogeneity (*I*^2^ = 57%). The pooled estimate was OR 0.45 (95% CI 0.34–0.61; *I*^2^ = 0%) among studies using threshold of ≤ 6 h. The single study using a threshold >6 h reported OR 0.03 (95% CI 0.00–0.47). The subgroup difference was borderline (*p* = 0.05). Because only three studies contributed and one subgroup contained a single study with a zero-event cell, this finding should be considered exploratory.

### Postoperative tracheostomy

**Figure 11:**
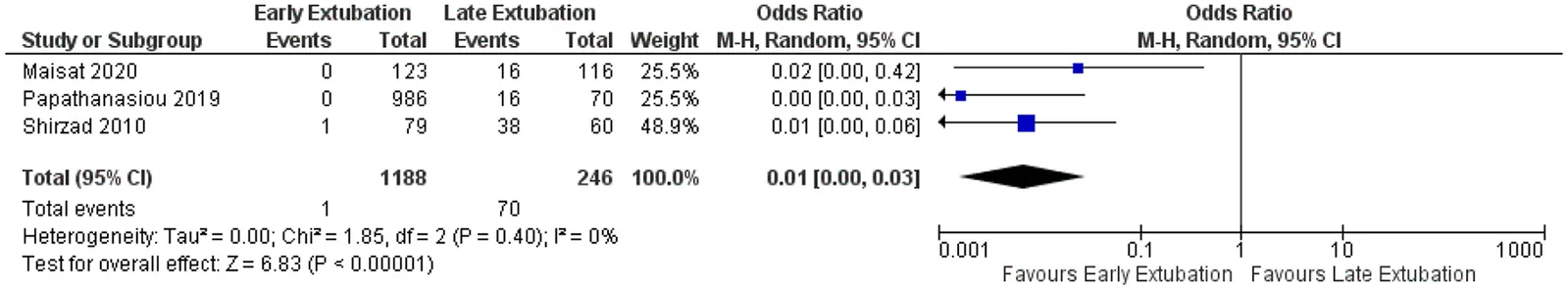
Early vs. Late Extubation, Outcome: post operative tracheostomy.

Figure 11 presents a forest plot comparing the incidence of postoperative tracheostomy between early and late extubation groups across three studies. The pooled odds ratio was 0.01 (95% CI: 0.00–0.03), indicating a highly significant reduction in tracheostomy rates among patients who underwent early extubation (*Z* = 6.83, *p* < 0.00001). All included studies Maisat (10), Papathanasiou (9), and Shirzad (11) reported near-zero or no tracheostomy events in the early group, in contrast to consistently higher rates in the late extubation cohort. For instance, Shirzad observed 38 tracheostomy cases among 60 late extubated patients vs. only one in the early group. No heterogeneity was detected (*I*^2^ = 0%, *p* = 0.40), underscoring the consistency of findings. These results suggest that early extubation is protective against the need for postoperative tracheostomy, likely by reducing the duration of mechanical ventilation and associated complications.

### Certainty of evidence (GRADE)

The certainty of evidence was assessed using GRADE (Table 2). Overall certainty was low to very low. The evidence was predominantly observational and was downgraded for selection bias, confounding by indication, reverse causation, inconsistent extubation definitions, heterogeneous operative populations, and imprecision. Statistical heterogeneity ranged from low for CRRT to extreme for ICU length of stay and mediastinal bleeding/re-exploration. Readmission was supported by only three studies and remained imprecise despite a statistically significant pooled association.

**Table 2:** GRADE summary of findings and certainty of evidence for primary postoperative outcomes.

Outcome	No. of studies	Study design	Certainty of evidence (GRADE)	Reason for downgrading
Mortality	12	Predominantly observational	⊕○○○ very low	Serious risk of bias and reverse causation; serious inconsistency (*I*^2^ = 86%); indirectness from heterogeneous thresholds and operations
ICU Length of stay	4 estimable	Observational studies	⊕○○○ very low	Serious risk of bias; extreme inconsistency (*I*^2^ = 100%); clinical heterogeneity; common measurement unit requires verification
Hospital length of stay	6 estimable	Predominantly observational	⊕○○○ very low	Serious risk of bias; serious inconsistency (*I*^2^ = 80%); indirectness; one study was not estimable
Postoperative pneumonia	7	Predominantly observational	⊕○○○ very low	Serious risk of bias and reverse causation; serious inconsistency (*I*^2^ = 78%); heterogeneous populations and definitions
Renal failure requiring CRRT	4	Observational studies	⊕○○○ very low	Serious risk of bias and reverse causation; indirectness; limited number of studies despite low heterogeneity (*I*^2^ = 18%)
Reintubation	7	Predominantly observational	⊕○○○ very low	Serious risk of bias; very serious inconsistency (*I*^2^ = 92%); imprecision and heterogeneous thresholds
Mediastinal bleeding/re-exploration	5	Predominantly observational	⊕○○○ very low	Serious risk of bias; extreme inconsistency (*I*^2^ = 97%); very serious imprecision and opposing study estimates
Tracheostomy	3	Observational studies	⊕○○○ very low	Very serious risk of bias and indirectness because tracheostomy is strongly determined by prolonged ventilation and postoperative course
Readmission	3	Observational studies	⊕○○○ very low	Serious risk of bias; moderate inconsistency (*I*^2^ = 57%); serious imprecision and one single-study subgroup

## Discussion

This systematic review and meta-analysis combined data from 19 observational and interventional studies comparing early and late extubation strategies in adult cardiac surgery patients. Earlier extubation was consistently associated with improved postoperative outcomes, including lower odds of mortality, reintubation, postoperative pneumonia, CRRT, tracheostomy, and hospital or ICU readmission, as well as shorter ICU and hospital length of stay. However, these findings should be interpreted with cautious because the available evidence mostly originates from observational studies. It is also important to highlight that some patients undergoing earlier extubation are generally more clinically stable and have fewer postoperative complications (12). Therefore, the observed benefits may reflect differences in patient characteristics rather than the effect of earlier extubation itself and should be interpreted in light of possible selection bias, confounding by indication, and reverse causation (13).

This systematic review evaluates early vs. late extubation across a broad range of cardiac operations among adult population. Although excluding pediatric studies improved population consistency, substantial clinical heterogeneity remained because the included studies include CABG, valve, combined, aortic, adult congenital, LVAD, and transplant procedures. These operations differ in complexity, cardiopulmonary bypass exposure, bleeding risk, hemodynamic support, and expected postoperative ventilation (14).

Our study showed that earlier extubation was associated with lower odds of reintubation and postoperative pneumonia. Reintubation was lower in the earlier-extubation group (OR 0.22, 95% CI 0.07–0.70), although heterogeneity was substantial (*I*^2^ = 92%). Postoperative pneumonia was also less frequent (OR 0.20, 95% CI 0.09–0.45; *I*^2^ = 78%). These findings do not indicate an increased reintubation risk among appropriately selected patients managed in accelerated recovery pathways. Nevertheless, they should not be interpreted as proof that earlier extubation independently prevents respiratory complications, because patients who develop instability, infection, or respiratory dysfunction are inherently more likely to remain ventilated.

Earlier extubation was associated with a substantially lower rate of postoperative tracheostomy, and this finding was consistent across the included studies (*I*^2^ = 0%). Patients with an uncomplicated postoperative recovery are more likely to be extubated early and less likely to require prolonged mechanical ventilation or tracheostomy. As tracheostomy is usually reserved for patients with persistent respiratory failure or other serious postoperative complications, the observed association is more likely to reflect differences in postoperative recovery than the effect of earlier extubation itself.

Earlier extubation was associated with a lower need for postoperative CRRT (OR 0.10, 95% CI 0.06–0.17; *I*^2^ = 18%). This can be explained by the fact that patients with positive postoperative recovery are more likely to be extubated earlier and less likely to develop severe acute kidney injury requiring CRRT (15). The low heterogeneity (*I*^2^ = 18%) suggests that this finding was consistent across the included studies. However, because most of the evidence came from observational studies, this association should not be interpreted as evidence of a direct causal effect.

In this study, we performed sensitivity analysis based on an early-extubation threshold of 6 h or less to align interpretation with contemporary fast-track practice and the 2024 joint ERAS Cardiac–ERAS International–STS consensus statement, which supports structured strategies facilitating extubation within 6 hours after routine elective cardiac surgery (6). However, some studies in this category used a 4-h cutoff. The subgroup should therefore be understood as a clinical threshold category rather than a uniform exact 6-h comparison. The consensus benchmark should also not be treated as a universal performance target for under-studied or complex populations such as ventricular-assist-device recipients, transplant recipients, and emergency surgical patients. In addition, the STS also reports prolonged postoperative intubation as a quality measure when ventilation exceeds 24 h; however, the publicly described NQF #0129 measure applies specifically to adults undergoing isolated CABG. It is, therefore, addresses a narrower population than the present review and should not be generalized uncritically to the growing spectrum of adult cardiac operations. The 6-h fast-track benchmark and the >24-h prolonged-ventilation metric answer different clinical questions and should be presented separately.

A pooled analysis of a completely homogeneous operative population was not feasible because most studies combined different cardiac procedures and did not provide procedure-specific outcome data. Consequently, residual heterogeneity cannot be fully controlled through conventional subgroup meta-analysis. Earlier extubation may also be a marker of uncomplicated recovery rather than the direct cause of improved outcomes, because clinical teams preferentially extubate patients who are physiologically stable and have experienced less complex perioperative courses. Future studies should stratify patients by baseline clinical risk and by the type and complexity of the surgical procedure, use standardized extubation definitions, report adjusted estimates, and provide procedure-specific outcomes.

Methodologically, the evidence base was dominated by retrospective observational studies. Although several studies performed adequately on the Newcastle–Ottawa Scale, uncertainty remained regarding cohort selection, exposure classification, outcome ascertainment, and adjustment for operative complexity and perioperative instability. Yigit et al. and Affronti et al. were judged to have higher overall risk of bias but remained represented in the available quantitative figures; their inclusion was therefore incorporated into the certainty assessment rather than described as an exclusion. The large pooled associations for mortality, CRRT, pneumonia, and reintubation are likely influenced by confounding by indication and reverse causation, because successful early extubation is itself a marker of an uncomplicated postoperative course.

### Clinical implications

The present findings support the use of structured early extubation pathways in carefully selected patients undergoing cardiac surgery. However, the available evidence does not establish that early extubation itself directly improves outcomes. Rather, successful early extubation appears to reflect appropriate patient selection, meticulous perioperative management, and multidisciplinary enhanced recovery protocols. Clinical decisions regarding extubation timing should therefore remain individualized rather than protocol-driven alone (16).

### Future directions

Future multicenter prospective studies and randomized evaluations of integrated fast-track pathways are needed. Analyses should be stratified by surgical complexity, operation type, baseline comorbidity, cardiopulmonary bypass duration, and anesthetic technique. Standardized extubation definitions and adjusted effect estimates are essential for developing patient-specific extubation algorithms. Longer follow-up assessing quality of life, functional recovery, and readmission would clarify the broader clinical impact.

### Limitations

This review has several important limitations. First, the majority of included studies were observational, making the findings susceptible to selection bias, confounding by indication, and residual confounding. Therefore, early extubation should be interpreted primarily as a marker of favorable postoperative recovery rather than an independent cause of improved outcomes. Second, considerable clinical heterogeneity existed across the included studies. Definitions of earlier extubation ranged across the studies included. In addition, surgical populations included CABG, valve, combined, aortic, adult congenital, transplant, LVAD, and mixed procedures. However, we performed Random-effects models to addressee between-study variability and performed sensitivity analyses based on extubation threshold (6 h or less or greater than 6 h) to investigate heterogeneity. However, procedure-specific subgroup analyses were not feasible because too few studies reported separable data. Third, several outcomes, including mediastinal bleeding, tracheostomy, and renal replacement therapy, are strongly influenced by baseline patient status and postoperative clinical course. Consequently, the observed associations should not be interpreted as evidence of causality. Finally, reporting of important perioperative variables was inconsistent across studies, precluding additional subgroup analyses and meta-regression. Specifically, cardiopulmonary bypass time, aortic cross-clamp time, and operative complexity were inconsistently reported across the included studies, preventing meaningful quantitative comparisons of these potential confounders. Although publication bias was assessed, small-study effects cannot be completely excluded. Consistent with the GRADE assessment, the certainty of evidence for most outcomes was low to very low because of the predominance of observational studies, substantial heterogeneity, and the potential for residual confounding.

## Conclusion

In adults undergoing cardiac surgery, earlier extubation was associated with favorable postoperative outcomes including lower mortality, shorter ICU and hospital stays, and lower rates of reintubation, postoperative pneumonia, tracheostomy, CRRT, and hospital or ICU readmission. However, future randomized trials and prospective studies are warranted as the available evidence was predominantly observational and clinically heterogeneous

## Data Availability

The original contributions presented in the study are included in the article/Supplementary material, further inquiries can be directed to the corresponding author.
