# Supplementary material for: Clinical outcomes of early vs. late extubation following cardiac surgery: a systematic review and meta-analysis
Source: Front Med (Lausanne). 2026 Aug 26;13:1913348. doi: 10.3389/fmed.2026.1913348 (PMC13553855; doi:10.3389/fmed.2026.1913348)
Supplement: Supplementary file 1 [file Supplementary_File_1.docx]

**Supplementary Table S1**. Extubation Definitions and Operative Characteristics of Included Studies

| **Study** | **Population** | **Cardiac Procedure(s)** | **Definition of Early Extubation** | **Definition of Late Extubation** |
| --- | --- | --- | --- | --- |
| Rashid et al., 2008 | Adult | CABG, valve replacement, ASR | <4 h | >4 h |
| Camp et al., 2009 | Adult | CABG, valve, CABG + valve | <6 h | >6 h |
| Thanavaro et al., 2021 | Adult | CABG, valve, combined CABG + valve | <48 h | >48 h |
| Fischbach et al., 2024 | Adult | Surgical aortic valve replacement (SAVR) | <6 h | >6 h |
| Zettervall et al., 2017 | Adult | EVAR/Open aortic repair | Immediate OR or <12 h | >12 h (EVAR); >24 h (open repair) |
| Naughton et al., 2003 | Adult | OPCAB, complex cardiac surgery | <6 h | >6 h, >24 h, >48 h |
| Yiğit et al., 2023 | Adult | CABG, valve, aortic dissection, LVAD/BIVAD | ≤8 h | >8 h |
| Aksoy et al., 2021 | Adult | Mixed cardiac surgery | <24 h | >24 h |
| Farooq et al., 2021 | Adult | CABG, valve, non-complex ACHD | ≤4 h | >4 h |
| Amini et al., 2022 | Adult | Mixed cardiac surgery | <6 h or 7–24 h | >24 h |
| Maisat et al., 2020 | Adult | Ascending aorta, arch replacement, CABG, AVR | ≤48 h | >48 h |
| Shirzad et al., 2010 | Adult | Valve surgery | <24 h | ≥24 h |
| Trouillet et al., 2009 | Adult | CABG, valve, transplant, aortic surgery | ≤72 h | >72 h |
| Papathanasiou et al., 2019 | Adult | LVAD implantation | ≤7 days ventilation | >7 days ventilation |
| Chan et al., 2018 | Adult | CABG, valve, combined surgery | ≤6 h | >6 h |
| Kern et al., 2001 | Adult | CABG, valve, combined surgery | <48 h | >48 h |
| Affronti et al., 2018 | Adult | Mixed cardiac surgery | ≤14 days | >14 days |
| Trouillet et al., 2011 | Adult (RCT) | CABG, valve, transplant, mixed cardiovascular surgery | Study protocol | Study protocol |
| Reddi et al., 2017 | Adult | CABG and/or valve surgery | <18 h | ≥18 h |
